# Exploring the importance of aromatic plants' extrafloral volatiles for pollinator attraction

**DOI:** 10.1111/nph.70496

**Published:** 2025-08-29

**Authors:** Aphrodite Kantsa, Consuelo M. De Moraes, Theodora Petanidou, Mark C. Mescher

**Affiliations:** ^1^ Department of Environmental Systems Science ETH Zürich Schmelzbergstrasse 9 8092 Zürich Switzerland; ^2^ Department of Geography University of the Aegean University Hill 81100 Mytilene Greece

**Keywords:** aposematism, bees, chemical ecology, ethnobotany, herbivory, Lamiaceae, leaves, terpenoids

## Abstract

Aromatic plants occur in many plant lineages and have widespread ethnobiological significance. Yet, the ecological significance and evolutionary origins of aromatic volatile emissions remain uncertain. Aromatic emissions have been implicated in defensive interactions but may also have other important functions. In this Viewpoint article, we propose an ecologically relevant definition for the aromatic phenotype and evaluate available evidence relating to the ecological role of aromatic emissions, focusing specifically on their role in pollinator attraction. We synthesize available literature addressing the use of extrafloral volatiles by pollinators, including evidence that aromatic plant emissions are primary foraging cues for some species, and present new behavioral findings documenting bee attraction to the aromatic lemon thyme in the absence of flowers. We highlight recent ecological research showing that aromatic species are highly influential in Mediterranean plant–pollinator communities and their emissions predict key interactions, particularly with bees. Based on the available evidence, we hypothesize that aromatic plants represent a form of chemical aposematism, wherein high levels of constitutive defense enable signaling phenotypes that convey information to both potential antagonists and mutualists. Finally, we outline future research priorities to clarify the role of aromatic emissions in information ecology and explore their application in agricultural systems.

## Περίληψη

Τα αρωματικά φυτά απαντώνται σε πολλές φυτικές γενεαλογικές γραμμές και έχουν ευρεία εθνοβιολογική σημασία. Ωστόσο, η οικολογική σημασία και η εξελικτική προέλευση των πτητικών εκπομπών τους παραμένουν αβέβαιες. Οι εκπομπές των αρωματικών φυτών έχουν συνδεθεί με αμυντικές διαειδικές αλληλεπιδράσεις, αλλά ενδέχεται να επιτελούν και άλλες σημαντικές λειτουργίες. Σε αυτό το άρθρο γνώμης, προτείνουμε έναν οικολογικά συναφή ορισμό για τον ‘αρωματικό φαινότυπο’ και αξιολογούμε τα διαθέσιμα δεδομένα σχετικά με την οικολογική σημασία των αρωματικών εκπομπών, εστιάζοντας ιδιαίτερα στον ρόλο τους στην προσέλκυση επικονιαστών. Συνθέτουμε τη διαθέσιμη βιβλιογραφία που εξετάζει τη χρήση εξωανθικών πτητικών εκπομπών από επικονιαστές, συμπεριλαμβανομένων ενδείξεων ότι οι εκπομπές αρωματικών φυτών λειτουργούν ως πρωταρχικά σήματα αναζήτησης τροφής για ορισμένα είδη. Παρουσιάζουμε επίσης νέα συμπεριφορικά ευρήματα που τεκμηριώνουν την προσέλκυση μελισσών στο λεμονοθύμαρο, ακόμη και απουσία ανθέων. Παραθέτουμε πρόσφατες οικολογικές μελέτες που δείχνουν ότι τα αρωματικά φυτά είναι είδη‐κλειδιά στις μεσογειακές κοινότητες φυτών–επικονιαστών και ότι οι πτητικές εκπομπές τους προβλέπουν βασικές διαειδικές αλληλεπιδράσεις, ιδιαίτερα με μέλισσες. Με βάση τα διαθέσιμα δεδομένα, διατυπώνουμε την υπόθεση ότι τα αρωματικά φυτά συνιστούν μια μορφή χημικού αποσηματισμού (aposematism), όπου τα υψηλά επίπεδα ιδιοσύστατων (constitutive) εκπομπών επιτρέπουν τη μετάδοση πληροφορίας τόσο σε πιθανούς ανταγωνιστές (χημική άμυνα) όσο και σε εταίρους αμοιβαιότητας. Τέλος, προτείνουμε κατευθύνσεις για μελλοντική έρευνα, με στόχο την αποσαφήνιση του ρόλου των αρωματικών εκπομπών στην οικολογία της πληροφορίας και να την αξιολόγηση των δυνατοτήτων εφαρμογής τους σε γεωργικά συστήματα.

## Resumo

As plantas aromáticas ocorrem em muitas linhagens vegetais e têm um significado etnobiológico generalizado. No entanto, o significado ecológico e as origens evolutivas das emissões voláteis aromáticas permanecem incertos. As emissões aromáticas têm sido implicadas em interações defensivas, mas também podem ter outras funções importantes. Neste artigo de opinião, propomos uma definição ecologicamente relevante para o fenótipo aromático e avaliamos as evidências disponíveis relacionadas ao papel ecológico das emissões aromáticas, com foco específico em seu papel na atração de polinizadores. Sintetizamos a literatura disponível que aborda o uso de voláteis extraflorais por polinizadores, incluindo evidências de que as emissões de plantas aromáticas são sinais primários de forrageamento para algumas espécies, e apresentamos novas descobertas comportamentais que documentam a atração das abelhas pelo tomilho‐limão na ausência de flores. Destacamos pesquisas ecológicas recentes que mostram que as espécies aromáticas são altamente influentes nas comunidades de plantas e polinizadores do Mediterrâneo e que suas emissões predizem interações importantes, particularmente com as abelhas. Com base nas evidências disponíveis, hipotetizamos que as plantas aromáticas representam uma forma de aposematismo químico, em que níveis elevados de defesas constitutivas viabilizam fenótipos de sinalização que transmitem informações tanto para antagonistas quanto para mutualistas potenciais. Por fim, delineamos as prioridades de pesquisa futuras para esclarecer o papel das emissões aromáticas na ecologia da informação e explorar sua aplicação em sistemas agrícolas.

## 摘要

芳香植物广泛分布于多种植物谱系中，并具有重要的民族生物学意义。然而，其芳香挥发物排放的生态意义及其进化起源仍不明确。已有研究表明，这些芳香挥发物参与防御性种间相互作用，但它们也可能具有其他关键生态功能。在本文中，我们提出了一个具有生态相关性的‘芳香表型’定义，并评估了现有关于芳香挥发物生态作用的证据，特别关注其在吸引传粉者方面的作用。我们综合了有关传粉者利用花外挥发物的文献资料，包括芳香植物挥发物作为某些物种主要觅食信号的证据，并呈现了新的行为学发现，表明蜜蜂在没有花朵的情况下也会被芳香的柠檬百里香吸引。我们强调了近期生态学研究的成果，这些研究显示芳香植物在地中海植物–传粉者群落中具有显著影响力，其挥发物能够预测与传粉者，尤其是蜜蜂之间的重要互作关系。基于现有证据，我们提出假设:芳香植物可能代表一种‘化学警戒’形式，即高水平的固有防御能力使其形成具有信号功能的表型，向潜在的拮抗者和互利者传递信息。最后，我们概述了未来的研究重点，以进一步阐明芳香挥发物在信息生态学中的作用，并探索其在农业系统中的应用潜力。.

*τῶν μὲν γὰρ τὸ ἄνθος μόνον χρήσιμον*

*καì τούτων τὸ μὲν εὔοσμον, ὥσπερ ìον, τὸ δ ἄνοσμον*,

*ὥσπερ διόσανθος φλόξ*.

*τῶν δὲ καì οἱ κλῶνες καì τὰ φύλλα καì ὅλως*

*ἡ πᾶσα φύσις εὔοσμος, οἷον ἑρπύλλου ἑλενίου*

*σισυμβρίου τῶν ἄλλων. ἄμφω δὲ φρυγανικά*».

*‘Of some [species] only the flower is useful; and of these, some are sweet‐scented, like violet, some scentless*,

*like carnation*.

*Of other [species], the branches and leaves, as well as their entire growth is fragrant, such as thyme, lesser calamint*, *bergamot‐mint and the others. Both groups occur in the phrygana’*. Theophrastus (4^th^ c. BC), *Historia Plantarum* 6.6.2

*«ἐν τούτῳ δὲ τῷ τόπῳ ἦν μὲν ἡ γῆ πεδίον ἅπαν ὁμαλὲς ὥσπερ θάλαττα*,

*ἀψινθίου δὲ πλῆρες· εἰ δέ τι καì ἄλλο ἐνῆν ὕλης ἢ καλάμου*, *ἅπαντα ἦσαν εὐώδη ὥσπερ ἀρώματα»*

‘*In this region, the ground was an unbroken plain, as level as the sea*, *and full of wormwood [*Artemisia absinthium*]; and whatever else there was on the plain by way of shrub or reed*, *was always fragrant, like spices*’ Xenophon (4^th^ c. BC), *Anabasis* 1.5.1

‘*Arbor aromatica*. *Aliquī aromaticitatem in cortice*. *Aliquī in flore*.’ ‘*The aromatic tree*. *Some [have] fragrant cortex*. *Some [have] fragrant flowers*.’ Bartholomæus Anglicus (13^th^ c. AD), *De Proprietatibus Rerum* XVII.II

«*…die ebenfalls wohlriechenden Blüten der […] Boronia elatior in den Kronenblättern überhaupt keine inneren Drüsen ausbilden, sondern in den Kelchblättern, und bei anderen Rutaceen, deren Laubblätter die Haberlandtschen Entleerungsapparate besitzen, in den Kronenblättern keine derartigen Einrichtungen nachweisbar sind*.» ‘*… the equally fragrant flowers of* Boronia elatior *[…] do not develop any internal glands in the petals at all, but in the sepals, and in other Rutaceae, whose leaves possess Haberlandt's secretory apparatus, no such devices can be detected in the petals*.’ Porsch (1906)

‘*A few strongly aromatic families like the Rutaceae, Lamiaceae, and Valerianaceae make an exception in that the flowers accumulate volatiles (and often the type of secretory apparatus) related to those of the vegetative body*.’ Vogel (1983)

‘*A walk through a conifer forest or a Mediterranean shrubland on a warm day provides convincing evidence that plants release substantial quantities of volatiles from vegetative as well as floral organs. However, the study of the physiology and function of these non‐floral perfumes is still in its infancy*.’ Pichersky & Gershenzon (2002)

## Introduction

As early as the 4^th^ century BC, the ancient Greek naturalist Theophrastus described a distinct category of plants characterized by strong constitutive volatile emissions from leaves and other vegetative parts (Theophrastus, 1916; Supporting Information Notes S1). However, the plant species typically identified as ‘aromatic’ are taxonomically diverse, and the term has no precise botanical definition. The volatile organic compounds (VOCs) released by vegetative parts of these plants are also diverse, frequently exhibiting complex blends rich in specialized terpenoids, including lineage‐specific mono‐, sesqui‐, and diterpenes (Pichersky & Raguso, 2018). A review examining 178 aromatic plants from Greece reported an average of 48 volatile VOCs in the essential oils of aerial parts, with species in the genera *Stachys* (Lamiaceae), *Anthemis, Achillea* (Asteraceae), *Cistus* (Cistaceae), and *Hypericum* (Hypericaceae) producing > 100 VOCs each (Kantsa *et al*., 2015). These VOCs are frequently bioactive, and aromatic plants have extensive ethnobotanical value in many cultures (Breitmaier, 2006; Bakkali *et al*., 2008; Wang *et al*., 2015; Pichersky & Raguso, 2018); moreover, aromatic species yield natural products with considerable and growing commercial value (Grand Review Research, 2020). Yet, the role of aromatic plants' volatile emissions (aromatic emissions hereafter) in natural biological communities is not well understood, and both the evolutionary origins of the aromatic phenotype and its ecological functions remain uncertain.

**Fig. 1:**
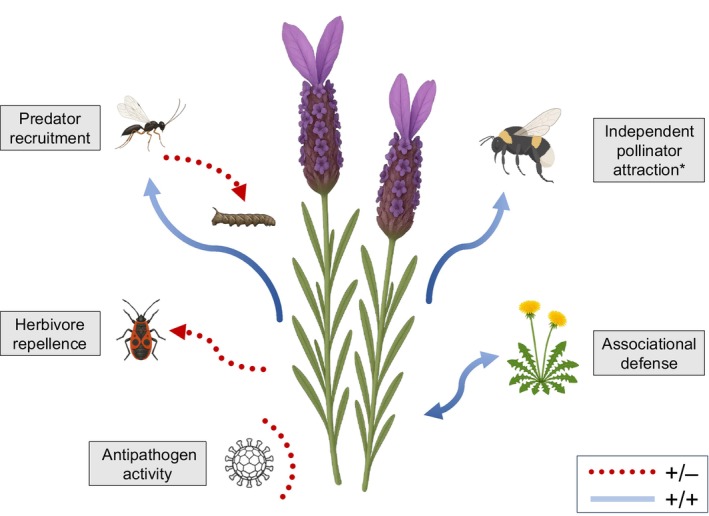
Known and proposed (*) ecological functions of aboveground aromatic emissions (i.e. constitutive volatile emissions (CVEs) from vegetative plant tissues) in mediating biotic interactions with arthropods, other plants, and microorganisms. Dashed red arrows indicate antagonistic interactions (+/−), and solid blue arrows indicate mutualistic interactions (+/+). The illustrations of species were created with ChatGPT‐4o (copyright‐free, AI‐generated) and subsequently verified against photographic material to ensure general botanical accuracy.

Ecological research on aromatic emissions has focused primarily on their role in interactions with herbivores and other plant antagonists (Fig. 1). Findings from this work provide evidence for herbivore repellence (Basedow *et al*., 2006; Hata *et al*., 2016; Li *et al*., 2020), direct toxicity (Kumar *et al*., 2011; Isman, 2020), attraction of herbivores' natural enemies (Wan *et al*., 2015; Togni *et al*., 2016; Batista *et al*., 2017; Hatt *et al*., 2019; Gong *et al*., 2024), and antimicrobial effects against phytopathogens (e.g. Lu *et al*., 2013). In addition, terpenoids from aromatic plants have been identified as allomones in the context of antagonistic (i.e. allelopathy) (Whittaker, 1970) and mutualistic (e.g. Karban *et al*., 2016) interactions between plants.

The hypothesis that aromatic volatiles might play a role in pollinator attraction was raised as early as the 1980s (Harborne, 1982; Vogel, 1983; Pellmyr *et al*., 1987; Meeuse, 1992), but was met with skepticism and somewhat vague arguments that attractive leaf emissions might disorient pollinators or interfere with chemical defense mechanisms (Pellmyr *et al*., 1987). Despite later evidence that vegetative volatiles can influence pollinator behavior, at least in synergy with floral volatile emissions (Raguso & Willis, 2005; Kárpáti *et al*., 2013), the hypothesis that vegetative emissions can act as independent attractants of pollinators has received little empirical investigation to date. Nevertheless, considerable indirect evidence indicates that this hypothesis deserves more attention. For example, the emissions of vegetative parts of aromatic plants frequently exceed those of flowers (Schultze *et al*., 1992; Keefover‐Ring, 2013), suggesting they might provide salient cues for pollinators over considerable distances, especially in environments in which aromatic species dominate the landscape, as they do in many Mediterranean communities (Pichersky & Gershenzon, 2002; Niemeyer & Teillier, 2007; Blondel *et al*., 2010).

In this Viewpoint article, we evaluate available evidence bearing on the potential role of aromatic emissions in plant–pollinator interactions. To facilitate evaluation of such evidence, we propose an ecologically relevant definition of the aromatic phenotype, which is essential for identifying the correct class of systems and interactions that need to be explained for resolving the origins and ecological significance of aromatic emissions. Using this definition, we discuss published findings that bear on the functional role of vegetative emissions in the context of plant–pollinator interactions. We also present new empirical results from behavioral experiments assessing bee attraction to foliar volatiles of the aromatic lemon thyme (*Thymus* cf. *citriodorus*). Based on these and other recent empirical findings, we suggest a potentially important ecological role for these emissions in plant–pollinator chemical communication. Finally, we propose research questions and priorities for future work that will elucidate the significance of vegetative volatiles in pollination.

## An ecologically relevant definition of aromatic plants

As noted, the term ‘aromatic’ has been used to refer to certain plants since antiquity (Anglicus, 1480; Porson, 1822; Kühn, 1830), often interchangeably with the term ‘medicinal’. While the designation is anthropocentric and largely subjective – the term ‘aromatic’ being derived from the Greek *ἄρωμα* (árōma), meaning ‘fragrance’ or ‘pleasant scent’ – it captures a striking characteristic of the chemical phenotype of plants spanning a wide range of taxa. However, the lack of a precise biological definition poses a challenge for efforts to explore the ecological roles of aromatic plants and the evolutionary pressures that give rise to their distinctive vegetative emissions. We therefore propose the following ecologically relevant definition, which we will utilize throughout this article:

*An aromatic plant is one that produces unusually high levels of volatile emissions constitutively (i.e*. *under typical, undisturbed conditions), from above‐ground vegetative tissues during at least some part of its lifecycle*.

The most significant divergence of the proposed definition from traditional usage is that it encompasses any plant species with high levels of constitutive vegetative emissions (CVEs, i.e. the baseline release of VOCs by healthy plants under non‐stressful conditions) relative to other plant species, independent of its economic or cultural significance for humans or the subjective human sensory experience of its scent. The traditional, anthropocentric framing can be biased by human standards (pleasantness, culinary use, or medicinal value), likely representing an incomplete sample of plants that exhibit ecologically relevant VOC emissions with adaptive significance. A nonanthropocentric approach is thus crucial for exploring the ecological functions of CVEs and broadens our focus to species not typically identified as aromatic, such as the intensely bitumen‐scented Arabian pea (*Bituminaria bituminosa*, Fabaceae) (Tava *et al*., 2007), the Eurasian wild garlic (*Allium ursinum*, Amaryllidaceae) (Kovačević *et al*., 2022), tomato (*Solanum lycopersicum*, Solanaceae) (Bautista‐Lozada & Espinosa‐García, 2023), liverworts (Marchantiophyta) (Wankhede & Manik, 2015; Asakawa & Ludwiczuk, 2018), or the Mediterranean dwarf palm (*Chamaerops humilis*, Arecaceae), which, as discussed below, attracts its pollinators with leaf instead of floral emissions (Dufaÿ *et al*., 2003). The inclusion of such species is also critical for exploring the potential selective pressures that may favor the evolution of signaling via aromatic emissions, potentially leading to convergent evolution across disparate plant taxa.

This reframing also allows us to avoid taxonomic assumptions and resolve ambiguities that arise when entire clades are classified as aromatic or nonaromatic without reference to the traits of individual species. Box 1 clarifies which trait combinations meet our criteria, with reference to specific taxa. Overall, our framework takes into consideration the plasticity of the volatile phenotype (Dicke, 2016) and the divergent selective pressures experienced across environments and evolutionary histories (Raguso, 2008, 2021), while avoiding utilitarian classifications. The resulting distribution of plants exhibiting aromatic phenotypes, according to our definition, spans diverse lineages, resonating with macroevolutionary patterns of ‘escape and radiate’ diversification proposed for highly biodiverse clades that frequently include taxa with high CVEs (Raguso, 2021). However, it is important to note that these lineages, such as the Labiatae, include also numerous nonaromatic taxa, and, conversely, aromatic taxa can occur in clades where they might be less expected, like Poaceae or Arecaceae (see Box 1 for details). Therefore, while macroevolutionary processes likely contribute to the prevalence of the aromatic phenotype in certain families, they do not fully account for its distribution across the angiosperms, reinforcing the need for an ecological, functional perspective to understand the evolution and adaptive significance of CVEs.

Box 1

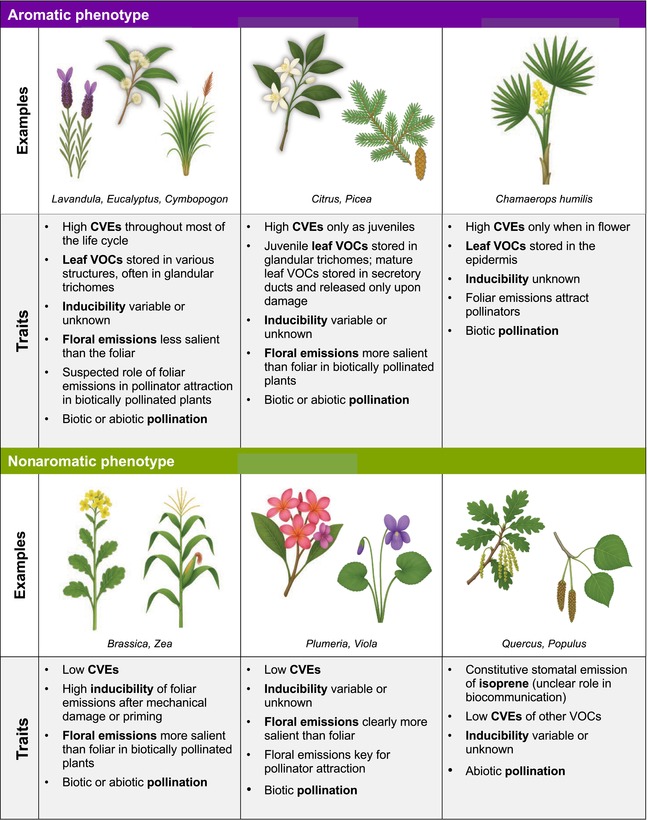

One limitation of the proposed definition is that it does not precisely describe what counts as a ‘high’ level of constitutive volatile emission – a threshold that may also shift across growth forms, taxa, and ecological scales. Given the pronounced scarcity of data documenting *in vivo* aromatic emissions (Kantsa *et al*., 2015) from the vast majority of angiosperm species, it remains unclear whether these plants constitute a special functional group of highly emitting species or, alternatively, the higher end of a phenotypic continuum of constitutive emission rates. The extent to which such emissions are associated with specific environmental selective agents (e.g. atmospheric or soil conditions, drought or other environmental stressors, or mutualistic or antagonistic interactions with other organisms) is also uncertain. The extensive literature on plant chemical ecology suggests that most plant species exhibit relatively low levels of CVEs, with higher emission rates – and qualitative changes in blend composition – being induced in response to biotic (e.g. herbivory and infection) and abiotic (e.g. drought or heat) stressors (Kessler, 2015). In addition to direct defensive and other functions, these induced emissions play a role in signaling interactions, including the recruitment of herbivores’ natural enemies (e.g. De Moraes *et al*., 1998). However, while there has been extensive work on the ecology of plant volatiles, large‐scale multi‐species studies of CVE phenotypes are rare (e.g. Sørensen *et al*., 2020; Losch *et al*., 2024). This is also true for many recognized aromatic species, whose high levels of emission are often inferred from direct sensory experience and essential oil analysis, rather than quantitative *in vivo* measurement. Consequently, as noted above, it is not currently possible to say whether aromatic species represent a truly distinct volatile emission phenotype.

Furthermore, it is also unclear whether the volatile signaling phenotypes of aromatic species exhibit convergent features beyond high levels of CVEs. While our definition focuses on the latter, the relationship of constitutive and induced emissions also has ecological relevance (e.g. Kariyat *et al*., 2012), and plant defense theories (reviewed by Koricheva *et al*., 2004) predict trade‐offs between constitutive and inducible defenses that may extend to defensive features of volatile signaling. Yet few studies have investigated the extent to which aromatic species exhibit inducible volatile responses, and the available evidence is inconclusive: in *Artemisia tridentata* (Asteraceae) and *Cymbopogon flexuosus* (Poaceae), certain terpenoids increased after mechanical damage (Kessler *et al*., 2006; Jiang *et al*., 2022); similarly, exposure of *Lavandula angustifolia* to MeJA led to shifts in volatile profiles (Dong *et al*., 2022); however, no such effect was found in *C. flexuosus* (Jiang *et al*., 2022) or in *Eucalyptus grandis* (Myrtaceae) (Henery *et al*., 2008). These variable results underscore the need for more comparative data on constitutive and induced volatile emissions across aromatic lineages to determine whether aromatic plants exhibit similarity in their overall volatile signaling phenotypes (i.e. comprising both constitutive and induced emission profiles).

Finally, it is important to note that the proposed definition is neutral with respect to the relative significance of diverse communicative functions (e.g. antagonism, mutualism) in driving the evolution of aromatic emissions, as well as the specific mechanisms plants use to produce these emissions, which, as discussed, may exhibit considerable variation across taxa. For example, extra‐floral VOCs can be produced in specialized (sub‐)epidermal structures of vegetative tissues, including glandular trichomes (e.g. in Lamiaceae, Asteraceae, Cannabaceae), idioblasts (e.g. Lauraceae), secretory cavities (e.g. Rutaceae, Myrtaceae), and secretory ducts (e.g. conifers, Anacardiaceae, Apiaceae) (Pichersky, 2020; Sousa *et al*., 2021) (see also Box 1).

## Empirical evidence that aromatic emissions mediate plant interactions with pollinators

Although the definition proposed in the previous section is neutral with respect to communicative functions of aromatic emissions, it provides a framework for evaluating these (or other) potential functions. This framework is particularly relevant for plant–pollinator interactions, as some of the clearest evidence for pollinator attraction (discussed below) comes from systems that have not traditionally been recognized as aromatic, and which have not been widely utilized by humans.

### Pollinator attraction by the Mediterranean dwarf palm

Direct empirical evidence of behavioral effects of vegetative plant odors on pollinators is available in at least one system involving a plant species classified as aromatic under our definition. Specifically, the pollinating weevil *Derelomus chamaeropsis* (Coleoptera: Curculionidae) was shown to be attracted to the foliar scent of the dioecious palm *C. humilis* (Dufaÿ *et al*., 2003). The leaves of this species are known to emit high levels of terpenoid‐dominated volatile blends (at rates 20‐ to 100‐fold higher than floral emissions) during anthesis. The exact structures in which the relevant compounds are produced and stored remain unknown, although the entire leaf appears to be scented (Caissard *et al*., 2004). Foliar volatile emissions are higher in male than female plants and peak with anthesis (Dufaÿ *et al*., 2004), strongly suggesting that foliar scents have evolved specifically for pollinator attraction in this system.

### New empirical findings: honeybee attraction of CVEs from lemon thyme

To our knowledge, no previous study has examined pollinator attraction to vegetative volatiles from a plant traditionally recognized as aromatic in the absence of flowers. Two previous studies reported pollinator attraction to leaves of aromatic species, *Majorana syriaca* (Lamiaceae) and *Lantana camara* (Verbenaceae), but only after preconditioning pollinators to foliar scents using artificial nectar, as these studies were focused on other hypotheses and not designed to specifically test attraction to foliar volatiles (Beker *et al*., 1989; Andersson & Dobson, 2003, respectively). To test the hypothesis that foliar volatiles of aromatic plants can attract pollinators independently of flowers, we performed dual‐choice behavioral experiments using honeybee workers (*Apis mellifera*) and the strongly aromatic plant lemon thyme (*Thymus citriodorus*, Lamiaceae).

**Fig. 2:**
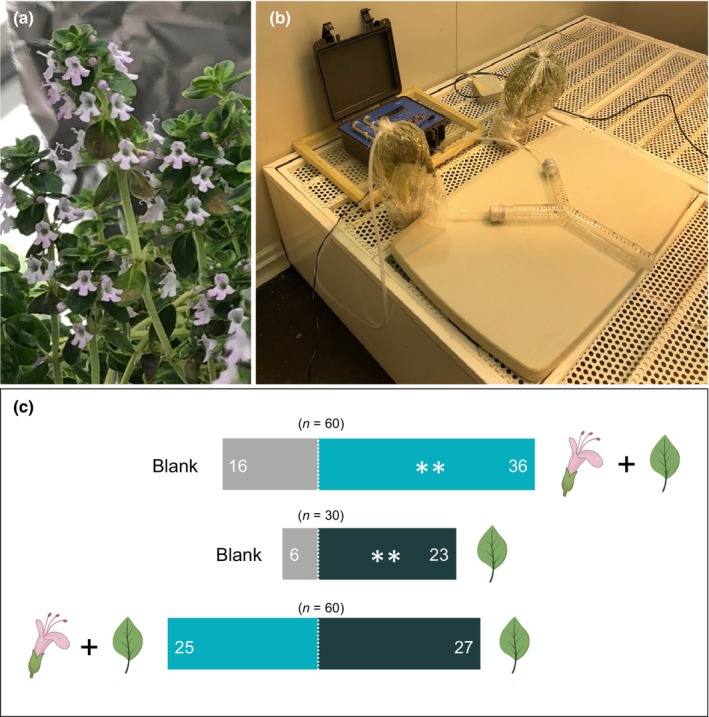
Honeybees are attracted to the volatile emissions of lemon thyme plants with and without flowers. (a) The inflorescence of lemon thyme. (b) Experimental setup: headspaces were covered with foil (not shown) to obscure visual cues. (c) Bee preferences in dual‐choice assays. Statistical significance was assessed with binomial tests (**, 0.001 < *P* < 0.01). Photos: A. Kantsa. Flower and leaf icons were created with ChatGPT‐4o (copyright‐free, AI‐generated) and subsequently verified against photographic material to ensure general botanical accuracy.

Our experiments assessed the behavioral responses of honeybee foragers to odors from plants in flower vs plants from which flowers had been removed as well as responses to each of these treatments vs clean‐air controls, using a Y‐olfactometer (see Notes S2 for details). The results showed that foragers with no previous experience of lemon thyme exhibited strong attraction to both flowering and flowerless thyme plants relative to controls. Moreover, when given a choice between plants, bees did not discriminate between the odors of plants in flower vs those from which flowers (minus the calyces) had been mechanically removed (Fig. 2). Furthermore, there was no significant difference across treatment comparisons in the time it took individual bees to make a choice.

These results indicate that vegetative volatiles emitted by thyme are attractive to honeybees; indeed, vegetative volatiles presented in isolation were no less attractive than the combined blend of vegetative and floral volatiles presented by plants in flower. Given the paucity of similar experimental work examining the effects of vegetative volatiles on pollinator behavior, this result suggests a need for additional studies, including work in natural settings. Future experiments might also investigate how repeated exposure to unrewarded vegetative cues might influence bee learning and modify foraging choices, illuminating the balance between innate attraction and experience‐dependent avoidance.

## Empirical evidence that aromatic plants are central species in plant–pollinator communities

Aromatic plant species are key components of many biological landscapes and can form extensive populations, as they frequently do in Mediterranean‐type ecosystems (e.g. Niemeyer & Teillier, 2007; Blondel *et al*., 2010). In the EU, for example, several officially designated habitat types are defined specifically by the presence of aromatic plant taxa (EU‐COM, 2013). While an inclusive meta‐analysis scrutinizing their role in natural interacting communities across ecosystems is lacking, there is evidence that aromatic plants (e.g. thyme, rockrose, lavender, oregano, germander) can be central nodes in plant–pollinator networks (Petanidou & Vokou, 1993; Kantsa *et al*., 2018; Nakas *et al*., 2023). In this context, centrality reflects how well connected and influential a plant species is within the overall network, combining both its direct interactions (closeness centrality) and its role in linking otherwise distant species (betweenness centrality) (Kantsa *et al*., 2018).

**Fig. 3:**
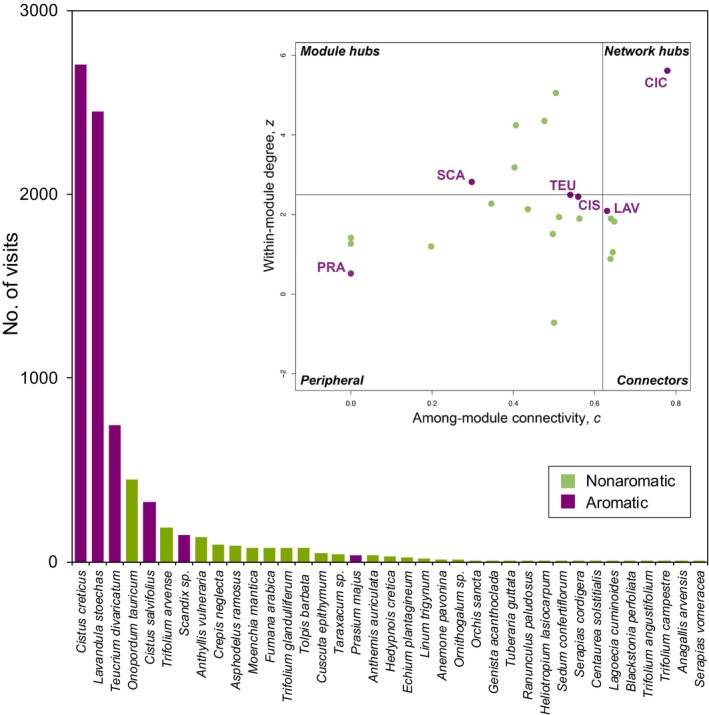
Pollinator visitation and functional roles of entomophilous species (*x*‐axis) during the spring/summer flowering season (April–July) in a Mediterranean‐type community. Inset: the functional roles of plant species (data points) in the plant–pollinator network, calculated *sensu* Olesen *et al*. (2007) (Supporting Information Notes S3). *Peripheral*, specialist plant species with only few connections to pollinators; *module hubs*, highly connected species within their own network compartment; *connectors*, species linking different network compartments; *network hubs*, highly generalist species, acting both as module hubs and connectors. CIC, *Cistus creticus* (Cistaceae); CIS, *C. salvifolius* (Cistaceae); LAV, *Lavandula stoechas* (Lamiaceae); PRA, *Prasium majus* (Lamiaceae); TEU, *Teucrium divaricatum* (Lamiaceae). For details on data and sampling methodology, see Kantsa *et al*. (2018).

Furthermore, there is evidence linking the centrality of aromatic plants in natural communities to features of their inflorescence emissions (including both petals and green parts). Specifically, in a Mediterranean scrubland community in Greece, aromatic plants were found to be the most‐visited plant species in the plant–pollinator network (Fig. 3), and this pattern was associated with inflorescence emissions, specifically mono‐ and sesquiterpenes. The latter were correlated with nectar presence and with floral color as perceived by bees, suggesting a potential signaling function. Sesquiterpenes were also positively associated with bee visitation, especially from the families Apidae and Megachilidae (the major pollinators in these environments), and with plant centrality within the pollination network, indicating that terpenoid‐dominated scents are an attribute of plant species that are most important for the structure of the community. Taken together, these results support the hypothesis that emissions from aromatic plants, and their role in mediating bee attraction, play a central ecological role in this Mediterranean ecosystem (Kantsa *et al*., 2017, 2018, 2019). Similar assessments in other Mediterranean‐type biomes around the world (Kantsa *et al*., 2023) could provide useful insights on the generality and context‐dependence of aromatic plant functions, given the similarities and differences in ecology and biogeography of these regions. Furthermore, targeted experimental manipulations – such as the removal or addition of dominant aromatic species within communities – could provide powerful tests of their specific ecological roles. Such approaches, analogous to experiments altering central plant species' presence (e.g. Maia *et al*., 2019; Bain *et al*., 2022), may offer direct insights into how aromatic plants shape plant–pollinator or plant–herbivore network architectures.

## Functional hypotheses for biocommunication

In addition to the direct evidence presented above, the hypothesis that foliar emissions play important roles in plant–pollinator interactions can be evaluated from a functional adaptationist perspective. Key questions here include whether the emissions of different aromatic taxa have similar and potentially convergent features that may be explained by similar signaling functions and, conversely, whether there are similarities in the ecology of aromatic species that might explain their similar emission phenotypes vis‐à‐vis other nonaromatic species.

### Functional advantages of foliar emissions for pollinator attraction

#### Oversized phenotypes and signal enhancement

One potential advantage for plants using vegetative volatiles to attract pollinators is the ability to produce higher levels of overall emission, which might enhance pollinator recruitment, particularly over long distances. So‐called ‘oversized’ phenotypic displays are known to enhance pollinator attraction, as reported in the tropical tree *Tibouchina pulchra* (Melastomataceae), where exceptionally large and densely growing flowers stand out against the green background of the Atlantic rainforest vegetation and provide effective visual cues for bees at up to 100 m (Brito *et al*., 2015). With respect to chemical cues, massive emissions from the entire aerial part of the plant may function in a similar way to recruit pollinators even over long distances. Moreover, previous findings (see the Empirical evidence that aromatic plants are central species in plant–pollinator communities section) indicate that inflated terpenoid emissions in aromatic plants can explain high visitation by bees and high centrality of these plants within the interacting community (Kantsa *et al*., 2017, 2018, 2019), supporting the hypothesis that vegetative emissions can enhance apparency to pollinators. At the community level, such exaggerated displays – whether visual or chemical – may also facilitate pollinator attraction by increasing overall resource detectability in diverse plant assemblages, though this remains an open question deserving targeted empirical research (Kantsa *et al*., 2017, 2018).

#### Signal persistence

Massive volatile emissions, especially of specific chemical classes, might also help to maintain signal efficacy in relation to abiotic conditions. For example, they may: facilitate the long‐distance dispersion of semiochemicals even where climatic conditions favor rapid evaporation (e.g. in hot and arid climates); or mitigate dilution of the blend by wind (e.g. Lawson *et al*., 2017). Thus, advantages arising from massive emission of hydrophobic volatile blends may contribute to the predominance of terpenoid compounds in the emissions of aromatic plants in Mediterranean‐type scrublands, where the climate is seasonally hot and dry and the low vegetation is exposed to wind (Whittaker, 1970; Pichersky & Gershenzon, 2002; Niemeyer & Teillier, 2007; Kantsa *et al*., 2018). Similar patterns may hold for floral scents, about which a recent global meta‐analysis revealed that climatic variables are significant predictors of the chemical composition of volatile blends, and that terpenoids are positively correlated with warmer and drier climates (Farré‐Armengol *et al*., 2020).

### Communication with antagonists and mutualists

As discussed above, most ecological research on aromatic plants has focused on the role of aromatic volatiles in interactions with herbivores and other plant antagonists. These functions provide an adaptationist explanation for the evolution of the aromatic emissions, independent of their potential role in pollinator attraction. However, these signaling functions need not be mutually exclusive; indeed, as suggested below, they may be complementary for aromatic species that are well defended against herbivory.

Previous work has highlighted trade‐offs in volatile signaling in the context of pollinator attraction and plant defense (e.g. via herbivore repellence or recruitment of natural enemies), especially in the context of induced volatile emissions: in such cases, volatiles that signal herbivore presence or plant defense status may also deter pollinators via changes in floral scent or convey information to pollinators that indicates reduced quality of floral resources (e.g. due to adverse effects of herbivory and defense induction) (Kessler, 2015). Aromatic species, by contrast, emit vegetative volatiles constitutively. This functioning may avoid trade‐offs with pollinator attraction, especially if high chemical apparency to herbivores is mitigated by strong constitutive defenses (Agrawal, 2007) that deter herbivory and thus prevent adverse impacts on floral resource quality. This suggests a scenario in which aromatic emissions that function, at least in part, as aposematic signals to herbivores (Woolfson & Rothschild, 1990), may simultaneously function in pollinator attraction.

Most research on volatile signaling in the context of plant–herbivore interactions has focused on the induced signaling phenotype and its role in herbivore repellence (De Moraes *et al*., 2001) and natural enemy recruitment (De Moraes *et al*., 1998), suggesting a common strategy that minimizes detection until defense is activated (Karban, 2011). For example, in *Solanum carolinense*, Kariyat *et al*. (2012) found that elevated CVEs due to inbreeding were linked to increased herbivory and reduced inducibility. This observation suggests the ‘typical’ plant volatile signaling phenotype pairs low levels of CVEs (to limit apparency to herbivores) with higher levels of induced emissions that function in direct and indirect defense. By contrast, aromatic plants may follow a distinct trajectory, combining high constitutive defense and CVEs with reduced inducibility and enhanced pollinator signaling. We hypothesize that this‘aromatic syndrome’ involves: high constitutive defense; high CVEs that convey information about defense status; and pollinator attraction via CVEs. Such a trajectory may parallel the evolution of aposematic signals in animals that later acquire a role in mate attraction, as seen in some brightly colored dart‐poison frogs (Cummings & Crothers, 2013).

As noted, we currently lack sufficient data to test whether aromatic plant species represent an entirely distinct signaling phenotype. However, this hypothesis challenges some previous arguments raised against a potential role for such emissions in pollinator attraction, including the initial skepticism based on concerns about signaling conflicts (Pellmyr *et al*., 1987). In this regard, it is also important to note that different ‘receivers’ of volatile information (individual species) may use different features of the volatile blend to construct ecologically relevant information specific to their own behavioral strategies (Mescher & Pearse, 2016); hence, there is not a single volatile communication ‘channel’ subject to competing constraints related to different receivers of volatile signals. Moreover, the specificity and reliability of signals may be further enhanced by multimodal cues or by temporal variation in emissions. For instance, via visual traits like the bracts of *Lavandula stoechas* (Lamiaceae) (Herrera, 1997) or through synchronized emission during floral development. The latter, although particularly important for confirming the pollinator‐directed signaling role in aromatic CVEs, has only been documented in the above‐discussed Mediterranean dwarf palm, *C. humilis*, where foliar emissions and flower bud maturation are known to be synchronized (Dufaÿ *et al*., 2004).

### Chemistry: terpenoids as key signaling compounds

Inferences about the potential signaling functions of aromatic emissions can also be drawn from the evaluation of their chemical composition. Terpenoid VOCs typically represent the most abundant and diverse chemical class in aromatic emissions: indeed, in a dataset of 174 aromatic taxa, 64% of the detected compounds (*n* = 999) were terpenoids (Kantsa *et al*., 2015). In other systems, these VOCs are frequently associated with defense purposes, even when emitted constitutively by flowers (Junker & Blüthgen, 2010; Schiestl, 2010; Pichersky & Raguso, 2018). Volatile terpenes also represent the second major class of inducible VOCs that are synthesized *de novo* (the other being the C6 green leaf volatiles) and are emitted systemically after damage (Holopainen, 2004). Moreover, terpenome diversity predicts herbivore–host association shifts within phylogenies (Becerra, 1997; Richards *et al*., 2015), and its remarkable complexity (Christianson, 2017) has often been attributed to a set of advantages regarding direct and indirect plant defense mechanisms (Table 1).

**Table 1:** Potential advantages of the complexity of terpenoid blends in plant defense.

Advantage	Mechanism
Increase efficacy of direct defenses	Synergistic effects among compounds
	Contingency among compounds (i.e. similar molecular structures but slightly different activities)
	Activity against multiple different enemies
Enhance indirect defenses	Increased information content for mutualists (e.g. parasitoids)
Optimize signal transmission	Properties favoring signal transmission and robustness under specific ecological and physical conditions
Disrupt enemies' adaptability^a^	Impede the evolution of counterdefense mechanisms by herbivores

Data have been combined from Gershenzon & Dudareva (2007), Gershenzon *et al*. (2012), and Pichersky & Raguso (2018).

^a^ Hypothesized for terpenoids here, based on findings from other compound classes.

Furthermore, specialized terpenoids represent a class of secondary metabolites bridging herbivory and pollination, showing diverse (context‐dependent) functionalities given that: they can be important bee‐produced semiochemicals (Rodriguez & Levin, 1976; Wheeler *et al*., 1977; Burge, 1980; Cane, 1986; Schiestl, 2010), thus possibly innate attractants; there is evidence that they (viz., monoterpenes) have secondarily evolved for attraction in newly radiated angiosperms (Schiestl, 2010); in the context of plant–pollinator communities, they are tightly linked with visual biases of bees, according to empirical evidence from a Mediterranean scrubland (Kantsa *et al*., 2017, 2018); and volatile terpenoids can attract generalist pollinators (e.g. honeybees; Laloi *et al*., 2000) or even highly specialized ones in floral emissions (Theis *et al*., 2014; Pichersky & Raguso, 2018), as well as parasitoids or predators when emitted by vegetative parts (Table 1). In this context, we hypothesize that terpenoid components of aromatic plants' CVEs represent key information‐conveying metabolites at least in environments like the dry and warm Mediterranean‐type ecosystems, where these plants form extensive populations and have been shown to be highly influential for the cohesion of entire communities (Petanidou & Vokou, 1993; Kantsa *et al*., 2018, 2019).

## Conclusions and future directions

Globally, stands of aromatic plants create rich and complex chemosensory landscapes. But, while humans have recognized these plants and made use of their unique chemical properties for millennia, we have a surprisingly limited understanding of the ecological functions of aromatic emissions or the environmental pressures that led to the convergent evolution of apparently similar chemical signaling phenotypes in disparate plant lineages. In this Viewpoint article, we have argued that CVEs in aromatic plants likely play a role in communication with pollinators, building on emerging evidence that their volatile profiles predict key interactions and influence within plant–pollinator communities, as well as evidence from previous studies and new data indicating that aromatic plants can attract pollinating insects even in the absence of flowers. We have also proposed an ecologically relevant definition of the aromatic phenotype that may facilitate further investigation on the role of this functional group of plants in this and other aspects of information‐mediated ecology. Finally, we have identified specific hypotheses and questions that bear on the proposed role of aromatic emissions in plant–pollinator interactions.

At the landscape level, we hypothesize that aromatic emissions may function in plant–pollinator interactions as enhancers of long‐distance signal strength and reliability under variable abiotic conditions, contributing to the prevalent role of aromatic species in plant–pollinator communities. We also propose a hypothesis regarding the evolutionary ecology of the aromatic signaling phenotype, in which high levels of CVEs are linked to high levels of constitutive plant defense and frequently play a dual role in plant communication with herbivores and pollinators, while avoiding the trade‐offs between defense signaling and pollinator attraction observed for herbivore‐induced volatiles. Consistent with these observations, we identify a number of open questions and priorities for future research. In overview, we suggest that future research efforts should focus on: characterizing the constitutive and induced volatile emissions of aromatic plants as well as patterns of emission that bear on functional hypotheses; exploring the phylogenetic and ecological variability of aromatic signaling phenotypes to identify patterns that may shed light on their evolution and ecological functions, as well as potential impacts of environmental change on signaling interactions (Borghi *et al*., 2019; Kantsa *et al*., 2023)—the potential role of aromatic emissions in cladogenesis may be of particular interest given their potential to simultaneously mediate ecological adaptation and reproductive interactions, making them good candidates for the identification of so‐called ‘magic traits’ that, under divergent selection, can also mediate nonrandom mating (Servedio *et al*., 2011); and directly assessing the role of aromatic emissions in pollinator attraction via laboratory and field experiments, particularly in the context of complex communities where plants engage in simultaneous interactions with mutualists and antagonists. Systematic comparisons of floral vs vegetative volatile emissions across diverse lineages would provide powerful tests of hypotheses on multimodal signaling and the potential role of pervasive CVEs as long‐range attractants complementing localized floral cues.

In addition to the fascinating natural history of aromatic plants, and the important role of aromatic emissions in natural communities, improved understanding of aromatic plant signaling phenotypes may also have practical implications, including in the context of sustainable agriculture. This is particularly the case in light of the potential role of aromatic emissions in both herbivore deterrence and pollinator attraction over long distances. Addressing the basic biological and ecological questions discussed in this article may therefore set the stage for future work exploring how aromatic plants, or relevant signaling traits, can be integrated into agricultural practices to enhance production and preserve biodiversity.

## Competing interests

None declared.

## Author contributions

AK and MCM conceived the idea; AK, CMDM and MCM planned and designed experiments; AK performed the experiments; TP hosted the experiments; AK analyzed the data; AK wrote the first draft; all authors contributed equally to revising the manuscript.

## Disclaimer

The New Phytologist Foundation remains neutral with regard to jurisdictional claims in maps and in any institutional affiliations.

## Supporting information


**Notes S1** Translations of ancient Greek texts.
**Notes S2** Behavioral assays.
**Notes S3** Species' functional roles in the pollination network.
Please note: Wiley is not responsible for the content or functionality of any Supporting Information supplied by the authors. Any queries (other than missing material) should be directed to the *New Phytologist* Central Office.
