# Supplementary material for: Exploring the importance of aromatic plants' extrafloral volatiles for pollinator attraction
Source: New Phytol. 2025 Aug 29;248(2):517–28. doi: 10.1111/nph.70496 (PMC12445819; doi:10.1111/nph.70496)
Supplement: Supplementary file 1 — Notes S1 Translations of ancient Greek texts. Notes S2 Behavioral assays. Notes S3 Species' functional roles in the pollination network. Please note: Wiley is not responsible for the content or functionality of any Supporting Information supplied by the authors. Any queries (other than missing material) should be directed to the New Phytologist Central Office. [file NPH-248-517-s001.docx]

## **New Phytologist Supporting Information**

**Article title:**

Exploring the importance of aromatic plants’ extra-floral volatiles for pollinator attraction

**Authors:**

Aphrodite Kantsa, Consuelo M. De Moraes, Theodora Petanidou, Mark C. Mescher

**Article acceptance date:**

22 July 2025

## **Notes S1: Translations of ancient Greek texts**

The English translation of aromatic species’ common names in the extract from *Historia Plantarum* follows Hort (1916) and has been confirmed using the works of Andrews (1958) and Scarborough (1984); for the non-aromatic species (violet and carnation), the translation was adapted by TP. The English translation of the extract from *Anabasis* is by Browson (1922).

## **Notes S2: Behavioral assays methodology**

Behavioral assays took place in a walk-in climatic chamber (Walk-in GRW-20 CMP3/TBLIN, CDR Chryssagis^TM^) simulating the local outdoor conditions during the experiments (T=25 ^o^C, H=50%). Experiments took place in June-July 2018 at the University of the Aegean on Lesvos Island, Greece. Pest-free *Thymus citriodorus* plants were commercially acquired from a nursery. Honeybee workers were collected in the morning before each experiment, at 07:00-07:30 am, from a beekeeping facility located nearby the university campus, using a handheld vac/aspirator (BioQuip, Rancho Dominguez, California). The bees were collected with the aspirator upon exiting their hives, they were transported in special ventilated vials, and they were left in the chamber for 30 min for acclimatization, during which time, they were offered artificial nectar. The bees never had visual contact with the experimental plants in the chamber.

Four sets of dual choice assays were performed, in which honeybees had to choose between: (i) floral vs. vegetative scent, (ii) floral scent vs. blank, (iii) vegetative vs. blank, and (iv) blank vs. blank, the latter to test for any unpredicted bias on the two branches of the Y-tube olfactometer. For the “floral” treatments, we left the plants intact, whereas for the “vegetative” treatment, we mechanically removed the flowers minus the calyces. The positions of the differently treated plants on the sides of the olfactometer were swapped randomly among the days of experiments, although the control assay (blank vs. blank) showed that there was no right/left bias (Exact binomial test, *P*=0.625). For each comparison, 60 different individuals were tested in total, except for the “vegetative vs. blank” experiment, where 30 bees were used. Each bee individual was tested only once and then removed from the experiment, precluding the possibility of within-assay learning or behavioral adjustment based on repeated unrewarded encounters.

Charcoal-cleaned air was pushed through the branches the olfactometer (600 mL min^-1^) after passing through the experimental headspaces made of polyethylene terephthalate oven bags (see Figure 2 in the Main Text). During the experiment, headspaces were externally covered so that bees could only rely on chemical cues. After acclimatization, each honeybee worker was left at the opening of the olfactometer, and its movement was monitored for 90 sec. First choices were recorded, as well as the time the bees needed to decide. Results were analyzed using exact binomial tests (R function *binom.test*).

## **Notes S3: Calculation of species’ functional roles in the pollination network**

To calculate the functional roles of plant species in the pollination network of the Mediterranean community studied in Kantsa *et al.* (2018), we used the methodology of Olesen *et al.* (2007), which is based on the implementation of a Simulated Annealing (SA) algorithm created for the study of metabolic networks (Guimerà & Nunes Amaral, 2005) and has ever since widely used for the study of the architecture of interspecific interaction networks. The SA algorithm calculates two species-level properties, i.e., the *within-module degree* (z) and the *between-module connectivity* (c), which can describe both the relative position of each species within the network, and how well it is connected with the other species and network compartments (modules). Olesen *et al.* (2007) distinguished four topological roles of species, based on the values of the two metrics. A **peripheral species** is characterized by both a low within-module degree (z ≤ 2.5) and low between-module connectivity (c ≤ 0.62). Such species maintain only a few interactions within their own module and have little to no connections with other modules. In contrast, a **connector species** also has a low within-module degree (z ≤ 2.5) but a high between-module connectivity (c > 0.62), linking different modules together and thereby contributing to the overall connectivity of the network. A **module hub** exhibits a high within-module degree (z > 2.5) but low between-module connectivity (c ≤ 0.62), indicating its central role in structuring interactions within its own module. Finally, a **network hub** has high values for both metrics (z > 2.5 and c > 0.62), reflecting its importance in maintaining coherence across the entire network as well as within its own module.

## **References**

**Andrews AC. 1958.** The Mints of the Greeks and Romans and Their Condimentary Uses. *Osiris* **13**: 127-149.

**Browson CL. 1922.** *Xenophon, Anabasis*. Cambridge, MA: Harvard University Press.

**Guimerà R, Nunes Amaral LA. 2005.** Functional cartography of complex metabolic networks. *Nature* **433**(7028): 895-900.

**Hort AFB. 1916.** *Theophrastus: Enquiry into plants and minor works on odours and weather signs*. London: William Heinemann.

**Kantsa A, Raguso RA, Dyer AG, Olesen JM, Tscheulin T, Petanidou T. 2018.** Disentangling the role of floral sensory stimuli in pollination networks. *Nat Commun* **9**(1): 1041.

**Olesen JM, Bascompte J, Dupont YL, Jordano P. 2007.** The modularity of pollination networks. *Proceedings of the National Academy of Sciences of the United States of America* **104**(50): 19891-19896.

**Scarborough J. 1984.** Early Byzantine Pharmacology. *Dumbarton Oaks Papers* **38**: 213-232.
